# Assembling the thymus medulla: Development and function of epithelial cell heterogeneity

**DOI:** 10.1002/bies.202300165

**Published:** 2023-12-31

**Authors:** Kieran D. James, Emilie J. Cosway, Sonia M. Parnell, Andrea J. White, William E. Jenkinson, Graham Anderson

**Affiliations:** ^1^ Institute of Immunology and Immunotherapy University of Birmingham Birmingham UK

**Keywords:** Aire, thymic epithelial cell, thymocytes, thymus

## Abstract

The thymus is a unique primary lymphoid organ that supports the production of self‐tolerant T‐cells essential for adaptive immunity. Intrathymic microenvironments are microanatomically compartmentalised, forming defined cortical, and medullary regions each differentially supporting critical aspects of thymus‐dependent T‐cell maturation. Importantly, the specific functional properties of thymic cortical and medullary compartments are defined by highly specialised thymic epithelial cells (TEC). For example, in the medulla heterogenous medullary TEC (mTEC) contribute to the enforcement of central tolerance by supporting deletion of autoreactive T‐cell clones, thereby counterbalancing the potential for random T‐cell receptor generation to contribute to autoimmune disease. Recent advances have further shed light on the pathways and mechanisms that control heterogeneous mTEC development and how differential mTEC functionality contributes to control self‐tolerant T‐cell development. Here we discuss recent findings in relation to mTEC development and highlight examples of how mTEC diversity contribute to thymus medulla function.

## Graphical abstract

The thymus medulla fosters self‐tolerant T‐cell development. We discuss recent findings in relation to medullary thymic epithelial cell (mTEC) development and highlight examples of how mTEC heterogeneity contributes to thymus medulla function, such as thymus trafficking and presentation of self‐antigens by promiscuous gene expression or through mimicking peripheral cells (mimetics).

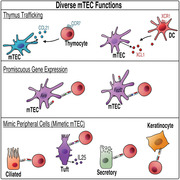

## INTRODUCTION

**FIGURE 1:**
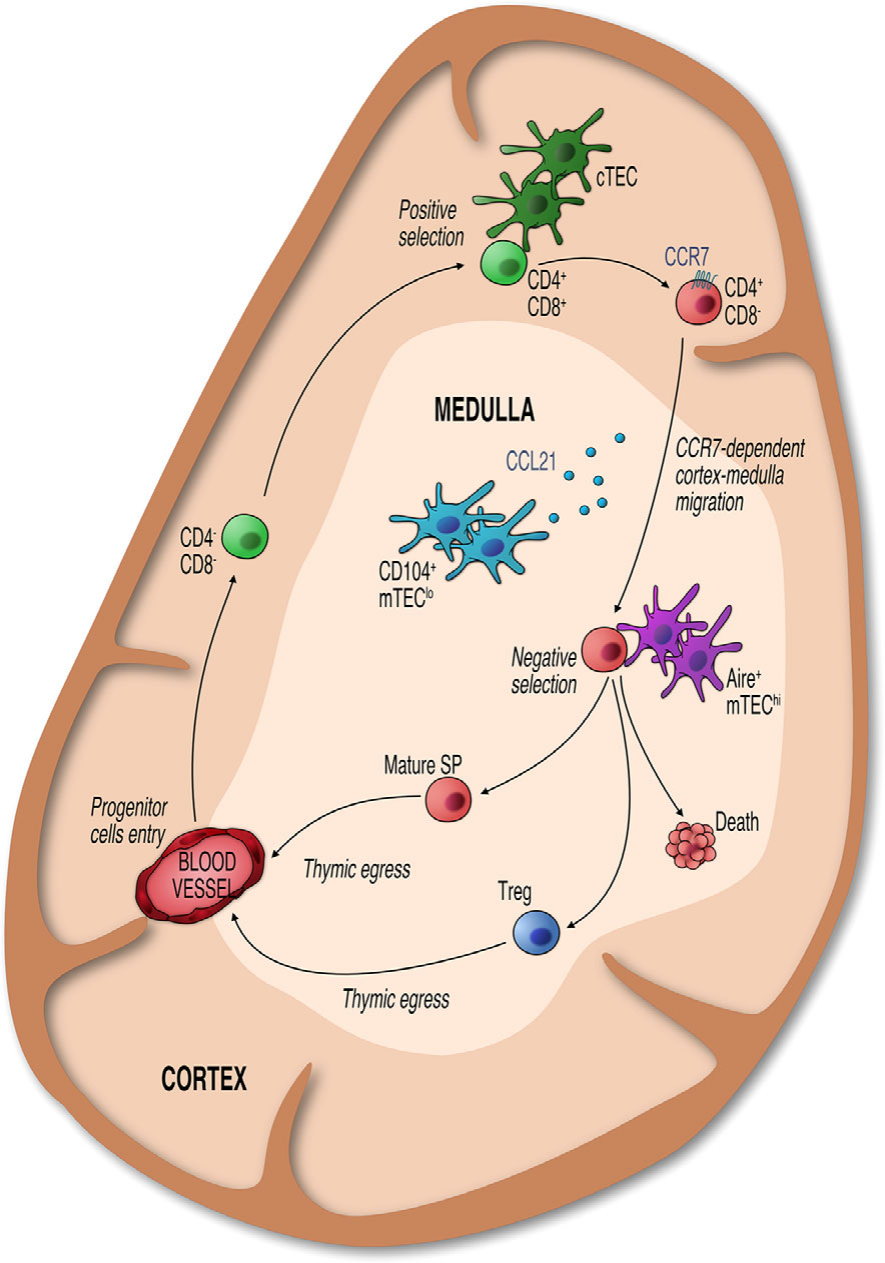
Organised Epithelial Microenvironments Support Intrathymic T‐cell Development. The adult thymus is organised into distinct outer cortical and inner medullary areas. In the cortex, cTEC control the maturation of CD4−CD8− progenitors entering via blood vessels at the cortico‐medullary junction. Following the generation and positive selection of CD4+CD8+ thymocytes, expression of CCR7 guides newly selected thymocytes into medullary areas, where interactions with mTEC and DC mediate tolerance induction, which includes the negative selection of cells bearing high affinity TCRs, and lineage divergence that results in the generation of Foxp3+ Treg. Following thymic selection, mature thymocytes exit the thymus at the corticomedullary junction and enter the peripheral T‐cell pool.

The thymus is a primary lymphoid organ anatomically located in the superior mediastinum.^[^ ^1^, ^2^ ^]^ Functionally, it tightly regulates T‐cell development, thereby ensuring the establishment of T‐cell‐dependent immunity that can contribute to effective immune responses. The major T‐cell subset that are generated intrathymically are conventional αβT‐cells, each endowed with the potential to express a single T‐cell receptor (TCR) specificity capable of recognising a limited array of antigenic peptides presented by self‐Major Histocompatibility Complex (MHC) molecules. The production of the αβTCR occurring during intrathymic T‐cell maturation involves random recombination of *Tcra* and *Tcrb* gene segments. While the advantage of random TCR generation ensures the production of a diverse TCR repertoire that can mediate immunologic protection against the multitude of potential antigenic challenges that may be encountered throughout life, it is also counterbalanced by the prospect of generating either non‐functional TCR specificities, or functional TCRs that are reactive against self‐antigen and possess the potential to drive autoimmune disease if left unchecked. To mitigate these unwanted outcomes of TCR generation, the developing αβTCR repertoire undergoes selection events to ensure that T‐cells exported into the periphery after intrathymic development are both functional yet self‐tolerant, and thereby capable of discriminating self from non‐self or altered‐self. To ensure self‐tolerant T‐cell production, highly specialised stromal microenvironments are present within the thymus. Grossly, the thymus is anatomically and functionally separated into two compartments, an outer cortex and inner medulla (Figure 1), both of which include thymic epithelial cells (TEC) ^[^ ^3^, ^4^ ^]^ and assorted non‐epithelial stroma cells such as mesenchymal cells.^[^ ^5^, ^6^ ^]^ The cortex operates to support early T‐cell development maturation through a process of bi‐directional signalling between the developing T‐cells (thymocytes) and cortical thymic epithelial cells (cTEC). These developmental events include T‐cell lineage commitment, leading to the specification and generation of CD4^−^CD8^−^ double negative (DN) thymocytes, which initially undergo TCRβ chain rearrangement and β‐selection, leading to subsequent upregulation of CD4 and CD8 co‐receptors to generate CD4^+^CD8^+^ double positive (DP) thymocytes and rearrangement of TCRα chain. A key role of cTEC is to present self‐peptide/MHC complexes against which developing DP thymocytes are able to test their randomly generated αβTCRs.^[^ ^7^, ^8^, ^9^ ^]^ Here, DP thymocytes capable of self‐peptide/MHC recognition are rescued from cell death through the process of positive selection (recently reviewed here ^[^ ^10^ ^]^). DP thymocytes that are able to successfully recognise αβTCR‐self‐peptide/MHC complexes presented by cTEC downregulate either CD4 or CD8 co‐receptor to become single positive (SP) thymocytes dependent on the capacity of their TCR to recognise self‐peptide loaded MHC class I or MHC class II molecules respectively.^[^ ^11^ ^]^

Following positive selection, SP thymocytes migrate from the cortex to the medulla to further continue their intrathymic development.^[^ ^12^, ^13^, ^14^ ^]^ This post‐positive selection stage of thymocyte maturation is supported and regulated by the complex intra‐medullary microenvironment consisting of multiple subtypes of medullary thymic epithelial cells (mTEC), dendritic cells, mesenchymal, and endothelial cells. Within the medulla, developing thymocytes undergo a series of post‐positive selection events, perhaps most notable of which is negative selection. Negative selection is a critical mechanism involved in the enforcement of central tolerance, contributing to the removal of T‐cell clones whose randomly generated αβTCR possess self‐reactive potential. Here, auto‐reactive thymocytes that possess the capacity to recognise self‐peptides presented on self‐MHC with a high level of affinity are removed from the developing thymocyte pool via the induction of apoptosis (Figure 1). Having passed through the developmental checkpoint of negative selection, the majority of surviving SP thymocytes complete their intrathymic development and emigrate from the thymus as naïve MHC class II‐restricted CD4^+^ or MHC class I‐restricted CD8^+^ αβT‐cells.^[^ ^15^, ^16^, ^17^ ^]^ In addition to naïve αβT‐cell generation, the medulla fosters the formation of immunosuppressive CD4^+^ Foxp3^+^ regulatory T‐cells (Treg) that act to maintain peripheral immunologic homeostasis, at least in part, by regulating the activity of autoreactive T‐cells that may have evaded intrathymic negative selection. The development of thymic Treg is driven by the interaction of the αβTCR with self‐peptide/MHC at an affinity higher than that of positively selected conventional naïve SP thymocytes but below the threshold for negative selection.^[^ ^18^, ^19^, ^20^ ^]^ A key consideration in the process of central tolerance is: How are developing thymocytes screened for autoreactive potential against self‐antigens associated with peripheral organs and tissues before they enter the periphery and drive unwanted tissue damage, whilst at the same being anatomically confined to the thymus for a relatively short duration? Critically, specialized subsets of mTEC, including those defined by expression of the autoimmune regulator (*Aire*) gene, possess the capacity to ectopically express a diverse array of peripheral tissue antigens (PTAs).^[^ ^21^, ^22^ ^]^ This, at least in part, endows the medulla with the capacity to negatively select developing autoreactive thymocytes, leading to deletion of clones expressing αβTCRs with high affinity to self‐antigens which could otherwise contribute to autoimmune disorders. Such self‐reactive T‐cells are triggered to undergo cell death resulting in their removal from the developing thymocyte pool.

Interestingly, the enforcement of T‐cell tolerance through negative selection appears not to be absolute, with studies indicating that this process may be leaky with autoreactive T‐cell clones being able to evade intrathymic deletion and enter the peripheral repertoire.^[^ ^23^, ^24^ ^]^ To help mitigate this potential outcome, Tregs function in a potentially dominant manner to prevent self‐reactive conventional T‐cells which have escaped negative selection and are therefore important for maintenance of tolerance to self in the periphery, as demonstrated by the autoimmune defects arising when Tregs are absent in both humans and in mice.^[^ ^25^, ^26^, ^27^, ^28^ ^]^

The functional capacity of mTECs to regulate multiple aspects of thymocyte development is underpinned by a spectrum of heterogeneity in the mTEC compartment, that at least at postnatal stages is maintained by a bipotent epithelial progenitor pool biased towards development of heterogenous medullary epithelial lineages.^[^ ^29^ ^]^ mTEC are made up of phenotypically and functionally distinct subsets which allows them to influence T‐cell development at multiple stages. As highlighted above, Aire is a key functional molecule expressed by a subpopulation of mTEC. Interestingly, not all mTEC express the transcriptional regulator Aire and not all PTAs are Aire‐dependent. Aligning with this, the transcriptional regulator FEZ family zinc finger 2 (Fezf2) controls an array of PTAs that are distinct from those dependent on Aire.^[^ ^30^ ^]^ Further, although some mTEC co‐express Aire and Fezf2 (Aire^+^ Fezf2^+^), analysis of adult mouse thymus also indicates the presence of Aire^−^ Fezf2^+^ subsets highlighting potential heterogeneity in the regulation and expression of PTA in mTEC via distinct mechanisms.^[^ ^31^ ^]^ In addition to mTEC heterogeneity with regard to PTA, the regulation of cortical to medullary thymocyte migration is driven by a specific subset of CD104^+^CD80^lo^MHCII^lo^ mTEC which produce the chemokine CCL21 to direct chemotaxis of post‐positive selection CCR7‐expressing CD4^+^ and CD8^+^ thymocytes towards and into the medulla.^[^ ^14^, ^32^, ^33^ ^]^ Recent advances in the definition of mTEC heterogeneity have contributed to the identification of numerous mTEC subsets which has expanded an understanding of how the medulla supports the development of varied T‐cell populations. With this review, we aim to provide an overview of aspects of mTEC heterogeneity within the adult thymus and discuss how differential mTEC subsets contribute to thymic function.

## EARLY CONCEPTS IN MTEC HETEROGENEITY

Initial studies analysing mTEC compartments of the adult murine thymus, commonly identified as EpCAM‐1^+^ TEC which bind the lectin UEA‐1 (EpCAM^+^UEA‐1^+^), reported two distinct mTEC sub‐populations based on the differential expression of MHCII and the co‐stimulatory molecule CD80. mTEC with low expression of CD80 and MHCII were termed mTEC^lo^, while CD80^hi^MHCII^hi^ mTEC were termed mTEC^hi^.^[^ ^34^ ^]^ Initial work in the embryonic thymus demonstrated that mTEC^lo^ appeared before mTEC^hi^ indicating a potential developmental progression of mTEC from mTEC^lo^ to mTEC^hi^.^[^ ^35^, ^36^ ^]^ Using an in vitro reaggregate thymic organ culture (RTOC) technique, a system where disaggregated embryonic thymic lobes can be mixed with sorted cell populations and then cultured, the developmental potential of mTEC^lo^ were investigated. In these experiments, isolated mTEC^lo^ cells that were cultured in RTOC gave rise to mTEC^hi^, presenting direct evidence that the mTEC^lo^ compartment contains precursors of mTEC^hi^.^[^ ^36^, ^37^ ^]^

mTEC^hi^ were thought to largely comprise a mature mTEC subset due to their differentially high expression of key functional molecules involved in tolerance induction of developing thymocytes, such as Aire, CD80, and MHCII, and high levels of promiscuous gene expression.^[^ ^22^ ^]^ Promiscuous gene expression, which enables mTEC expression of PTAs, is important for negative selection, the process where thymocyte clones bearing TCRs with a high affinity to self‐antigens undergo deletion. To achieve this, PTAs expressed by mTEC can be presented either directly or indirectly via transfer to intrathymic dendritic cells and subsequent presentation to developing thymocytes.^[^ ^38^, ^39^, ^40^ ^]^ Those thymocytes which do not recognise or interact with PTAs presented via self‐MHC with a high level of affinity may continue their development, and those thymocytes possessing a degree of affinity below the threshold for negative selection being potentially selected for regulatory T‐cell (Treg) development.^[^ ^41^ ^]^ The promiscuous gene expression driving representation of PTAs in thymus allows mTEC to express over 80% of the protein‐coding genome, resulting in the presentation of a diverse range self‐antigens to developing thymocytes.^[^ ^42^, ^43^, ^44^ ^]^ It has been reported that each mTEC expresses a mosaic of PTAs, however only 1%–3% of mTEC express a particular PTA, but importantly a single mTEC may express upwards of 300 different PTAs.^[^ ^43^, ^44^ ^]^

Further contributing to the idea of mTEC^hi^ as a functionally mature mTEC subset was the identification of Aire expression in this medullary epithelial subset, the expression of which is a key regulator of the capacity of mTEC to express a defined array of the PTAs expressed within the thymus.^[^ ^21^, ^22^, ^45^ ^]^ The importance of Aire's role in intrathymic PTA expression is demonstrated via the observation that mutations in Aire can lead to targeted autoimmunity against Aire‐dependent self‐antigens. For example, Aire‐deficient mice are reported to exhibit multi‐organ autoimmunity characterised by autoantibodies and lymphocytic infiltrates against target tissues including the liver, pancreas, salivary gland and testis,^[^ ^21^, ^46^ ^]^ correlating with autoimmune features characteristic of Autoimmune polyendocrinopathy‐candidiasis‐ectodermal dystrophy (APECED) in human patients driven by mutations in *AIRE*.^[^ ^47^ ^]^ In line with high CD80, MHCII, and PTA expression, within the thymic epithelial compartment expression of Aire was found to be restricted to mTEC^hi^, highlighting the importance of this cellular subset in central tolerance induction.^[^ ^22^, ^34^ ^]^ It should be noted however that, in addition to expression in mTEC fractions, Aire has also been found to be expressed within additional thymic‐resident cell types including thymic B cells.^[^ ^48^ ^]^ Interestingly, further to playing a critical role in regulation of PTA expression by mTEC that facilitate negative selection, Aire is additionally involved in promoting the generation of glucocorticoids that conversely promote thymocyte survival and potentially oppose negative selection indicating that Aire^+^ mTEC potentially influence thymocyte selection and repertoire via multiple distinct and complementary mechanisms.^[^ ^49^ ^]^

An additional example of the role of heterogenous mTEC in regulation of thymocyte maturation and tolerance is demonstrated by the capacity of Aire^+^ mTEC^hi^ to produce the chemokine XCL1, which acts to coordinate the localisation of XCR1 expressing conventional DC type 1 subsets (cDC1) within the thymic medulla.^[^ ^50^ ^]^ This appears to be involved in coordinating transfer of mTEC‐derived self‐antigens to intrathymic DC, a mechanism where mTEC co‐opt DCs to expand the spread of self‐antigen by cross‐presentation. cDC2 and plasmacytoid dendritic cells (pDC), in a similar fashion to cDC1, possess the capacity to interact and acquire self‐antigen from mTEC.^[^ ^51^, ^52^ ^]^ However, whilst cDC1 are predominantly involved in cross presentation of mTEC‐acquired antigen, cDC2 and pDC also present self‐antigen obtained from the periphery,^[^ ^53^, ^54^ ^]^ with CCR2^+^ cDC2 being recruited into the thymus in response to CCL2 produced by mTEC.^[^ ^55^, ^56^, ^57^ ^]^ Collectively, these mechanisms may be of importance in contributing to the enhancement of efficiency for tolerance enforcement particularly given the relatively short medullary dwell time of single positive thymocytes estimated to be in the region of 4–5 days.^[^ ^58^ ^]^

Aire^+^mTEC^hi^ were also found to be post‐mitotic, arising from cycling mTEC^lo^ and were concluded to be a terminally differentiated population, perhaps suggesting a final or late stage of mature mTEC development.^[^ ^37^ ^]^ It is important to note that whilst Aire‐deficient mice have altered PTA expression within mTECs, medullary PTA expression is not completely defective and mTEC still express Aire‐independent PTAs.^[^ ^22^, ^59^ ^]^ In part, the Aire‐independent expression of PTAs is driven by the transcriptional regulator Fezf2.^[^ ^30^ ^]^ Similar to deficiency in Aire leading to a breakdown in aspects of central tolerance and manifestation of targeted autoimmunity, Fezf2 expression by mTEC also plays an essential role in enforcing self‐tolerance. Fezf2‐deficient mice also demonstrate autoantibodies and inflammatory cell infiltration in organs including brain, kidney, liver, and salivary gland, and interestingly the patterns of autoimmunity in Fezf2‐ and Aire‐deficient mice were reported to be non‐overlapping indicating a cooperative role of these pathways and the heterogenous mTEC‐defined by their expression in the regulation of central tolerance.

## DEVELOPMENTAL COMPLEXITY IN THE MTECLO COMPARTMENT

Although the demonstration that mTEC^lo^ can generate mTEC^hi^ implies the presence of precursors within this population, it is now clear that mTEC^lo^ do not simply exist as a transitory, immature development stage prior to the formation of mTEC^hi^. Indeed, studies revealed that the mTEC^lo^ compartment is a highly complex population which contains a mixture of functionally and developmentally distinct subsets. A key example of this was the identification of mTEC^lo^ as the thymic stromal cell source of CCL21, a chemokine ligand for the chemokine receptor CCR7.^[^ ^33^ ^]^ Importantly, CCL21 has been shown to control the migration of positively selected thymocytes into the medulla (Figure 1),^[^ ^14^ ^]^ and the migration of cDC1 dendritic cell precursors into the thymus.^[^ ^60^ ^]^ Together with mTEC^hi^ expression of XCL1,^[^ ^50^ ^]^ these findings also highlight the notion that both mTEC^lo^ and mTEC^hi^ serve as distinct essential sources of chemokines, required to direct the migration and positioning of different cell types within the thymus.

The mTEC^lo^ compartment has also been shown to include cells which had at one stage in their history been Aire‐expressing mTEC^hi^, commonly referred to as post‐Aire mTEC. Evidence that Aire^+^ mTEC^hi^ may not be a terminally differentiated “end‐stage” population and may indeed undergo further development, came from observations from *Aire^−/−^ mice*, where the frequency of keratin 10 (Krt10)^+^ mTECs and highly‐keratinized terminal‐stage involucrin^+^ Hassall's corpuscles were significantly reduced ^[^ ^61^ ^]^. This potentially fits with the finding that Aire not only contributes to expression of PTAs via direct transcriptional regulation, but also indirectly by controlling mTEC heterogeneity.^[^ ^62^ ^]^ The appearance of both Krt10^+^ mTEC and Hassall's corpuscles occurs after the development of Aire^+^ mTECs during ontogeny,^[^ ^63^ ^]^ suggesting a precursor‐product relationship between Aire^+^ cells and Krt10^+^/Involucrin^+^ cells. Indeed, cell fate mapping experiments demonstrated that Aire^+^ mTEC could develop further into an Aire^−^ mTEC population expressing intermediate levels of MHC Class II.^[^ ^64^ ^]^ Additional experiments by Wang et al (2012) also described similar findings and highlighted that expression of Aire is required for the development of post‐Aire mTEC, as in the absence of Aire there is a reduction in post‐Aire mTEC.^[^ ^65^ ^]^ Interestingly, the developmental regulation and composition of mTEC^lo^ fractions including pre‐Aire mTEC precursors and post‐Aire subsets are critically regulated by self‐reactive CD4^+^ thymocytes that influence mTEC^lo^ transcriptional programmes and thereby influence the development and maintenance of their own selecting microenvironments.^[^ ^66^ ^]^ Collectively, such findings show that mTEC^lo^ are highly diverse, containing progenitors of mTEC^hi^, functionally mature CCL21^+^ mTEC, Fezf2^+^ mTEC, and post‐Aire mTEC highlighting the significance of understanding mTEC^lo^ heterogeneity and their developmental and functional relationship to their mTEC^hi^ counterparts.

The question of which cells within the mTEC^lo^ fraction give rise to mTEC^hi^, and additionally whether there are distinct mTEC^lo^ cells which give rise to functional CCL21^+^ mTEC^lo^ cells that are not post‐Aire mTEC still remains unclear. Relevant to this, Onder et al reported that Podoplanin^+^CD80^−^Aire^−^ mTEC located at the corticomedullary junction were mTEC restricted progenitors, suggesting the usefulness of Podoplanin expression to delineate mTEC progenitors within mTEC^lo^.^[^ ^67^ ^]^ Recently, studies using scRNA‐seq and trajectory analyses aimed to define the possible relationships between Aire^+^ mTEC, CCL21^+^ mTEC, and a proliferating mTEC cluster referred to as proliferating, or transit‐amplifying, mTEC.^[^ ^68^, ^69^, ^70^ ^]^ While data indicated that proliferating mTECs may be a precursor population to both CCL21^+^ and Aire^+^ mTEC subsets, it is still unclear how CCL21^+^ mTEC and Aire^+^ mTEC arise in the adult mouse thymus.^[^ ^70^ ^]^ Further characterisation of the heterogeneity and developmental kinetics of mTEC^lo^, mTEC^hi^ and post‐Aire mTEC, devised a new approach to define mTEC^hi^ into subsets based on their expression of the cell surface markers Sca‐1 and CD24.^[^ ^71^ ^]^ The authors defined three subsets of mTEC^hi^, mTEC^A/hi^ (CD24^−^Sca‐1^−^, mTEC^B/hi^ (CD24^+^Sca‐1^−^), and mTEC^C/hi^ (CD24^+^Sca‐1^+^). mTEC^A/hi^ included mostly Aire^+^ cells, mTEC^B/hi^ was a mixture of both Aire^+^ and Aire^−^ cells, and mTEC^C/hi^ were mainly Aire^−^. Transcriptomic analysis of these populations revealed that A, B, and C mirrored the specific genetic program of early, late, and post‐Aire mTECs. Consistent with a post‐Aire mTEC phenotype, mTEC^C/hi^ downregulated their expression of MHCII, CD80, PTAs, and most importantly Aire.^[^ ^71^ ^]^ Using cell surface markers to identify different stages within the transition from Aire expressing mTEC^hi^ to post‐Aire mTEC development opens the opportunities to investigate this process using techniques beyond sequencing.

## POST‐AIRE STAGES IN THE MTEC LINEAGE

Further understanding of mTEC heterogeneity has been supported by studies undertaking single cell RNA sequencing analysis of adult TEC compartments.^[^ ^72^, ^73^, ^74^, ^75^, ^76^, ^77^, ^78^ ^]^ Importantly, two studies in 2018 highlighted surprising heterogeneity within mTEC^lo^. Bornstein et al subdivided mTEC into four functionally distinct subsets: “mTEC I” expressed low levels of MHCII, CCL21 and were also CD104^+.[^ ^73^ ^]^ While mTEC II resembled mTEC^hi^ and expressed the highest levels of Aire, mTEC III expressed markers associated with post‐Aire mTEC, such as Krt10. Finally, the subset labelled mTEC IV did not express typical mTEC or cTEC markers, but instead expressed a gene signature and morphology closely associated with epithelial cells of the gut known as tuft cells. As such, these cells are commonly referred to as thymic tuft cells and can be identified by their expression of genes such as *Dclk1* and *L1cam*, with approximately 10% of mTEC being reported to be DCLK1 bright.^[^ ^73^ ^]^ Thymic tuft cells were also described simultaneously by Miller et al, who also used an inducibly labelled Aire fluorescent reporter mouse model to study mTEC heterogeneity.^[^ ^77^ ^]^ The authors sorted four populations of mTEC based on MHCII and Aire expression, identifying “Pre Aire” (MHCII^lo^ RFP^low^), Early Aire‘ (MHCII^hi^ RFP^low^), “Late Aire” (MHCII^hi^ RFP^hi^) and “Post Aire” (MHCII^lo^ RFP^+^). As expected, Aire expression and PTA expression was highest in the early and late Aire expressing cells. However, two distinct transcriptional signatures were identified in the post‐Aire subset, one which was enriched for markers of cornified epithelial cells such as Krt10, with the other resembling tuft cells. Importantly, engraftment of tuft cell deficient *Pou2f3^−/−^* thymus into athymic Nude mice revealed that thymic tuft cells play a key role in tolerance induction, whereby absence of thymic tuft cells, that represent an exclusive source of intrathymic IL‐25, led to the generation of an IL‐25 targeted auto‐antibody response demonstrating the functional potential of these specialized mTEC to contribute to self‐tolerance.^[^ ^77^ ^]^ Further investigation of thymic tuft cell development has revealed that in line with promotion of tuft cell maturation in peripheral tissues, thymic tuft cells have been reported to be co‐ordinately dependent on the transcription factor Sox4 indicating that such highly differentiated mTEC may utilise similar overlapping transcriptional pathways as their specialised peripheral counterparts.^[^ ^79^ ^]^ In addition, more recently the development of tuft cells in the thymus has been shown to be dependent on epigenetic modification mediated by Sirt6 highlighting the critical role of epigenetic regulators in the establishment of diverse mTEC compartments.^[^ ^80^ ^]^

## BEYOND THYMIC TUFT CELLS: THYMIC EPITHELIAL MIMETICS

**FIGURE 2:**
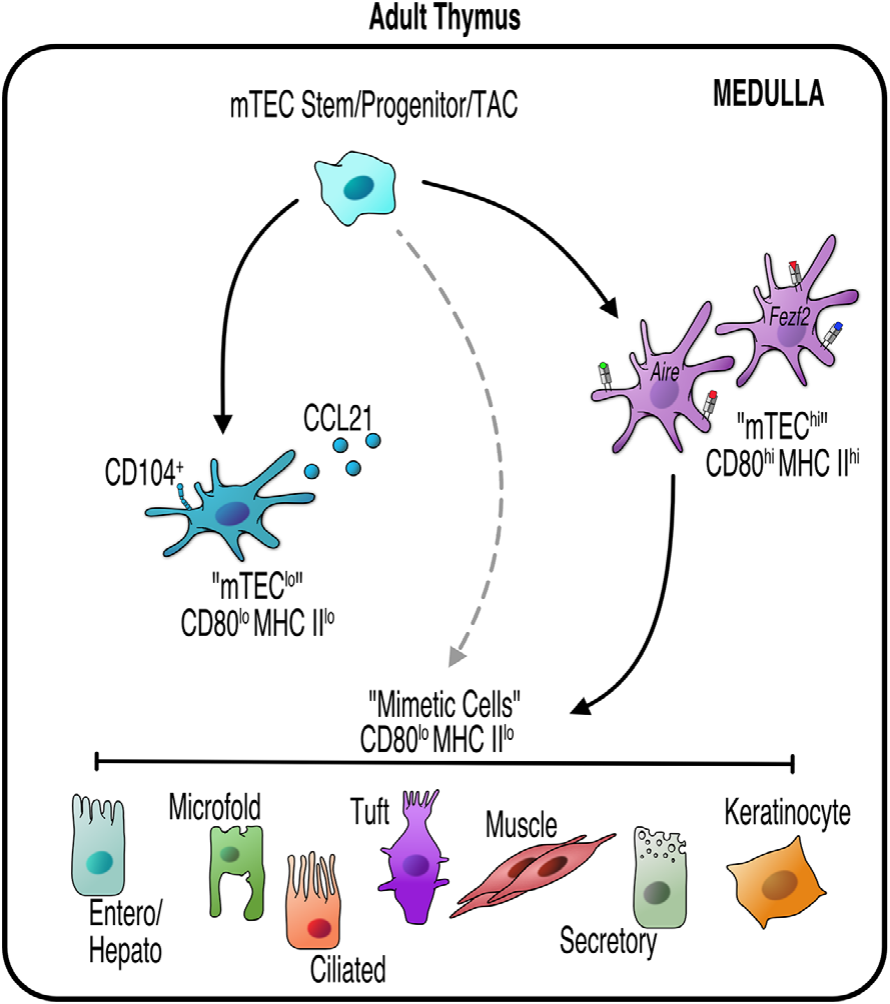
mTEC Diversity in The Adult Thymus. mTEC progenitors give rise to functionally distinct mTEC populations which can be identified by expression of low or high levels of MHC Class II (MHC II) and CD80. CCL21‐producing CD104^+^ mTEC^lo^ are essential in CD4^+^ and CD8^+^ thymocyte positioning. mTEChi which express the transcription factors Aire, and/or Fezf2, produce a diverse array of peripheral tissue antigens (PTAs) essential for tolerance. Recent advances have identified further heterogeneity within mTEClo, including thymic mimetics that are defined by expression of key cell‐specific transcription factors. Mimetic cells are thought to be post‐Aire (black solid line), however it has been shown that not all mimetic cells require Aire for their development, indicating a possible alternative route for their development (demonstrated by a grey dotted line).

The presence of cells within the mTEC population that possess transcriptional and phenotypic characteristics of peripheral tuft cells has recently been expanded considerably to show that other epithelial cell types typical of non‐thymic tissues are represented within the thymus. Described by Michelson and colleagues as thymic mimetic cells, these cells represent specific subsets of mTEC which mimic diverse extrathymic epithelial cell types including those present in skin, lung and liver, to expose developing thymocytes to a wide range of self‐antigens in a coordinated manner.^[^ ^69^ ^]^ This description of TECs sharing features with epithelial cells in non‐thymic peripheral tissues mirrors historical observations of morphological heterogeneity within the medulla. For example, in discussing the idea of a “mosaic of self” in relation to immune tolerance mechanisms, Farr and Rudensky discussed the idea of PTAs being produced and presented through the representation of peripheral cell types within the thymus.^[^ ^81^ ^]^ In more recent studies, Michelson et al. used scATAC‐seq to investigate PTA expression within individual mTECs, and identified several distinct mTEC subtypes which appeared to be post‐Aire and enriched for lineage‐defining transcription factors of extra‐thymic tissues.^[^ ^69^ ^]^ Importantly, each of these post‐Aire clusters showed specific enrichment of lineage‐defining transcription factor motifs in their accessible chromatin which mirrored that of peripheral cell types. Importantly, tuft cells were among these clusters. The authors named each cluster after its peripheral counterpart, giving keratinocyte, ciliated, secretory/neuroendocrine, enterocyte/hepatocyte, microfold, and tuft mTECs, and grouped these mTECs under the term “mimetic cells” (Figure 2). The expression of the lineage‐defining transcription factors of different mimetic mTEC subsets was essential for their development in the thymus: mice lacking the tuft cell transcription factor *Pou2f3* lacked thymic tuft cells, while mice lacking the transcription factors *SpiB* or *Sox8* that regulate peripheral M cells lacked their corresponding thymic mimetic populations.^[^ ^69^, ^77^ ^]^

Additional studies have expanded on this initial characterisation of mimetic cells to examine the mechanisms driving mimetic cell differentiation and function, by focusing on entero‐hepato mimetic cells, an mTEC population which shares a transcriptional program with gut and liver epithelial cells.^[^ ^82^ ^]^ Importantly, the authors revealed a key mechanistic distinction of mimetic cell development. Mimetic cells do not convert from mTECs into their bona fide peripheral cell counterpart, but instead layer genomic and transcriptomic programs onto a core, retained mTEC identity, by accessing enterocyte chromatin and transcriptional programs via the enterocyte‐specific transcription factors Hnf4α and Hnf4γ.^[^ ^82^ ^]^ Complementary studies using multiomic approaches to develop an atlas of thymic epithelial heterogeneity have further characterised mTEC mimetic cells, including further revealing previously poorly defined subsets mimicking parenchymal endocrine and microfold cells that were found to be differentially dependent on expression of *Insm1* and *Spib*, that also respectively control the development of peripheral endocrine and M‐cells in a manner similar to that observed for *Sox4*‐dependent regulation of thymic tuft cell development.^[^ ^79^, ^83^ ^]^ Interestingly, the same studies uncovered that thymic microfold mimetics appear to possess functional similarities to M cells that reside in peripheral mucosal tissues, including a capacity to undergo reciprocal interactions with B cells leading to the induction of IgA^+^ thymic‐resident plasma cells.

**FIGURE 3:**
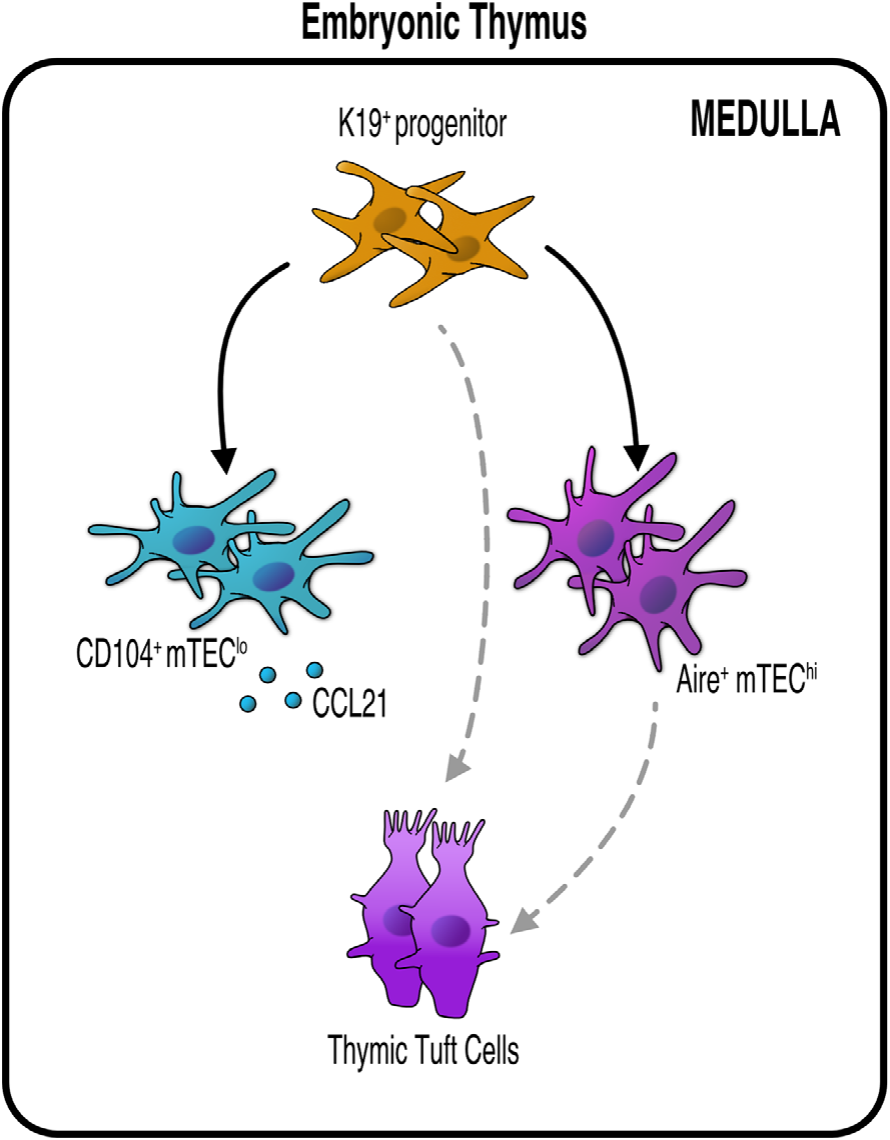
K19^+^ Multipotent Medullary Thymic Epithelial Progenitors Give Rise to Functionally Distinct mTEC Subsets in Embryonic Thymus. mTEC development involves a series of precursor‐product relationships that gives rise to the generation of multiple functionally distinct mTEC subtypes. In a recent study,^[^ ^84^ ^]^ we identified a multipotent mTEC progenitor (mmTECp) within the mTEClo compartment defined by expression of K19. K19^+^ mmTECp are mTEC‐restricted and capable of the long‐term production of multiple mTEC types, including Aire^+^ mTEC, thymic tuft cells, and CCL21^+^ mTEC^lo^. The ability of mmTECp to give rise to other mTEC subtypes, including additional thymic mimetic populations, requires further study.

To develop an understanding of the mechanisms that regulate development and maintenance of thymus mimetic cells, the maturation pathways and lineage relationships of such cells require further examination. Relevant to this, we recently identified an early precursor mTEC population defined by expression of cytokeratin19 (K19).^[^ ^84^ ^]^ Cell tracing experiments revealed that K19^+^ embryonic TEC can give rise to a diverse range of mTEC subsets, including CCL21^+^ mTEC^lo^, and Aire^+^ mTEC^hi^, suggesting a common origin for both cell types. Interestingly, K19^+^ progenitors also generated Dclk1^+^ thymic tuft cells, demonstrating their ability to give rise to at least one subset of thymus mimetic cells.^[^ ^84^ ^]^ While these studies identify a progenitor cell type in the thymus medulla that can generate functional diversity within the mTEC lineage (Figure 3), the ability of K19^+^ mTEC progenitors to give rise to additional thymus mimetics is currently not clear.

The discovery of mimetic cells builds on our understanding of PTA expression within the thymus and reveals that in addition to a quasi‐random expression of PTAs achieved by *Aire*, there are additional mechanisms that include a more coordinated regulation of PTA representation within medullary microenvironments. Here, mTEC mimic peripheral cells through expression of lineage‐defining transcription factors and adoption of peripheral cell characteristics to induce self‐tolerance. Thus, as well as understanding how mimetic cells differentiate in the thymus, it will also be important to determine the relative contributions of Aire^+^ mTEC and mimetic cells to PTA representation and their regulation of thymocyte maturation and central tolerance. *Aire* therefore plays a multi‐layered role in PTA expression as demonstrated by recent advances in understanding regulation of PTA expression by mimetic cells. For example, *Aire* is at least partially responsible for mimetic cell accumulation.^[^ ^69^ ^]^ Consistent with earlier work on keratinocyte‐like mTEC, *Aire*‐deficient mice have quantitative reductions in equivalent post‐Aire mTEC populations.^[^ ^65^ ^]^

## MTEC HETEROGENEITY FOSTERS DIVERSITY IN T‐CELL DEVELOPMENT

The presence of multiple mTEC subsets within the adult thymus medulla not only indicates a multi‐stage developmental programme for thymus medulla formation, but also suggests functional diversity within the mTEC compartment. For example, Miller et al described a role for tuft cells in regulating non‐conventional T‐cell subsets, namely Eomes^+^ SP8 and iNKT2 development through tuft cell production of IL25.^[^ ^77^ ^]^ Whilst initial iNKT development requires CD1d‐dependent interactions with CD4^+^CD8^+^ thymocytes in the cortex to produce CCR7^+^ iNKT progenitors,^[^ ^85^ ^]^ subsequent stages of iNKT‐cell development require further signals within the medulla.^[^ ^86^, ^87^ ^]^ Importantly, the functional definition of individual iNKT1, iNKT2, and iNKT17 subsets ^[^ ^88^ ^]^ has also enabled further investigation into the functional importance of the thymus medulla for iNKT‐cell development. For example, trans‐presentation of the cytokine IL15 by CD104^+^ mTEC^lo^ is important in the regulation of intrathymic iNKT1 and iNKT17 development, while IL25 production by thymic tuft cells controls iNKT2 development.^[^ ^32^, ^77^ ^]^ Thus, distinct mTEC subsets that reside within the CD80^lo^MHCII^lo^ population differentially regulate thymic iNKT populations.

As highlighted previously, in addition to enforcing central tolerance via the deletion of autoreactive T‐cell clones, the thymic medulla provides an essential developmental niche for the generation of Treg.^[^ ^89^ ^]^ Although the precise relative contribution of heterogenous mTEC subsets to Treg development is still to be fully appreciated, studies initially revealed that expression and presentation of a model PTA on Aire^+^ mTEC drives the development of antigen‐specific Treg.^[^ ^90^ ^]^ In addition to this, further studies investigating the importance of Aire, and mTEC presentation of PTA, on Treg repertoire have revealed that both direct presentation of antigen by mTEC is essential for the selection of a unique repertoire of Treg, and further that the presence of Aire^+^ mTEC is essential for shaping the Treg repertoire.^[^ ^91^ ^]^ Although Aire appears to play a critical role in determining Treg diversity, through use of RAG2GFP mice to discriminate newly generated Treg from their mature counterparts that have re‐entered the thymus from the peripheral circulation, it has been revealed that new Treg are generated at normal numbers in Aire‐deficient thymus. In contrast to the impact of Aire on quantitative de novo production of Treg, the thymus of Aire‐deficient mice demonstrates a reduction in the number of mature recirculating CCR6^+^ Treg as a consequence of reduced CCL20 production by mTEC^hi^.^[^ ^92^ ^]^ Of note, the Aire‐dependent recirculation of mature, peripheral Treg to the thymus is of functional relevance due to the potential of such cells to compete for intrathymic IL‐2 availability and thereby influence IL‐2 dependent intrathymic Treg development.^[^ ^93^ ^]^ Moreover, recent observations have further revealed that in addition to regulating the re‐entry of peripheral Treg to the thymus, Aire^+^ mTEC restimulate such recirculating cells and maintain their suppressive function and capacity to attenuate autoimmune disease.^[^ ^94^ ^]^

Although the role of Aire in Treg development has begun to be unravelled, the relative contribution of Fezf2 remains comparatively less well examined. Interestingly, the proportion of Foxp3^+^ Treg amongst SP4 thymocytes is reduced by approximately forty percent in the thymus of mice possessing a TEC‐specific deletion of Fezf2.^[^ ^30^ ^]^ Whether such a reduction in thymic Treg is attributable to a decrease in de novo Treg production versus a reduction in peripheral Treg recirculation back into the thymus, and indeed whether Fezf2 also impacts Treg TCR repertoire and restimulation as observed in the absence of Aire remains to be fully examined.

In addition to their influence on αβTCR‐expressing T‐cells, mTEC have been shown to regulate other non‐conventional T‐cells, including cells belonging to the γδT‐cell lineage. During embryogenesis, the thymus produces waves of distinct effector γδT‐cell subsets, and the development of the first wave involving the production of Vγ5^+^ thymocytes that exit the thymus to reside within the skin as dendritic epidermal T‐cells (DETCs). Interestingly, transplantation of *Relb^−/−^* thymus lobes that are devoid of mTEC failed to generate Vγ5^+^ thymocytes. Moreover, further experiments showed that Vγ5^+^ thymocytes express RANKL and aid in initial RANK‐mediated thymus medulla formation.^[^ ^95^ ^]^ Thus, crosstalk mechanisms are important in the control of both γδT‐cell and mTEC development. Within the adult thymus, we recently identified an essential role for mTEC in the development of effector IFNγ^+^ γδ‐T‐cells.^[^ ^96^ ^]^ In the absence of mTEC, the population of IFNγ‐producing γδ thymocytes was significantly reduced, whilst non‐effector cells were unaffected. Furthermore, absence of CCL21 chemokine production by mTEC, resulted in a similar loss of IFNγ‐producing γδT‐cells. These data indicate that as in the embryonic thymus, medullary microenvironments present within the adult medulla are essential in the development of effector γδ‐T‐cells. Further experiments aimed to examine the ability of the thymus medulla to control diverse T‐cell production in both fetal and adult life, will lead to a better understanding of the importance of this site for thymus function.

## CONCLUSIONS

Analysis of the mTEC compartment is an important area of thymic biology. Recent advances have seen significant progress in our understanding of the complex heterogeneity that exists within this population. As approaches for high throughput and multidimensional analyses become more accessible, our understanding of the complex nature of the mTEC compartment is likely to grow further. For example, the use of massively parallel flow cytometry, utilised in the recent study by Klein and colleagues, highlights opportunities and approaches to further probe mTEC heterogeneity.^[^ ^97^ ^]^ Further additional use of in vivo cell fate mapping approaches and in vitro RTOC systems will continue to be a means to study the developmental relationships of defined mTEC subsets.

As well as understanding mTEC heterogeneity under homeostatic conditions, exploring how mTEC heterogeneity is impacted in settings of dysregulated homeostasis is a further important area of future research. For example, recovery of thymus function following ablative therapy and reconstitution via bone marrow transplantation has been shown to result in the breakdown of central tolerance mechanisms caused by a failures mTEC regeneration.^[^ ^98^ ^]^ While the reasons for this failure are not yet clear, one possibility is that the frequency and/or developmental potential of mTEC progenitors, which may include K19^+^ multipotent mTEC progenitors ^[^ ^84^ ^]^ is impaired by pre‐conditioning ablative therapies such as radiation. Whatever the case, these findings highlight the importance of understanding the mechanisms that ensure correct generation of mTEC diversity, and perhaps most notably the identification and analysis of mTEC progenitors that give rise to functionally distinct mTEC subsets. Indeed, it is anticipated that a clearer understanding of mTEC development will ultimately aid in approaches to manipulate thymus function for future therapeutic benefit.

## AUTHOR CONTRIBUTIONS

The review was written by Kieran D. James and was revised by Emilie J. Cosway, Sonia M. Parnell, Andrea J. White, William E. Jenkinson, and Graham Anderson. All authors contributed to the article and approved the submitted version.

## CONFLICT OF INTEREST STATEMENT

The authors declare no conflicts of interest.

## Data Availability

Data sharing not applicable to this article as no datasets were generated or analysed during the current study.
